# Histone Acetyl Transferase 1 Is Essential for Mammalian Development, Genome Stability, and the Processing of Newly Synthesized Histones H3 and H4

**DOI:** 10.1371/journal.pgen.1003518

**Published:** 2013-06-06

**Authors:** Prabakaran Nagarajan, Zhongqi Ge, Bianca Sirbu, Cheryl Doughty, Paula A. Agudelo Garcia, Michaela Schlederer, Anthony T. Annunziato, David Cortez, Lukas Kenner, Mark R. Parthun

**Affiliations:** 1 Department of Molecular and Cellular Biochemistry, The Ohio State University, Columbus, Ohio, United States of America; 2 Department of Biochemistry, Vanderbilt University School of Medicine, Nashville, Tennessee, United States of America; 3 Department of Biology, Boston College, Chestnut Hill, Massachusetts, United States of America; 4 Ludwig Boltzmann Institute for Cancer Research (LBI-CR), Vienna, Austria; 5 Clinical Institute of Pathology, Medical University of Vienna, Vienna, Austria; University of Massachusetts Medical School, United States of America

## Abstract

Histone acetyltransferase 1 is an evolutionarily conserved type B histone acetyltransferase that is thought to be responsible for the diacetylation of newly synthesized histone H4 on lysines 5 and 12 during chromatin assembly. To understand the function of this enzyme in a complex organism, we have constructed a conditional mouse knockout model of Hat1. Murine Hat1 is essential for viability, as homozygous deletion of Hat1 results in neonatal lethality. The lungs of embryos and pups genetically deficient in Hat1 were much less mature upon histological evaluation. The neonatal lethality is due to severe defects in lung development that result in less aeration and respiratory distress. Many of the Hat1^−/−^ neonates also display significant craniofacial defects with abnormalities in the bones of the skull and jaw. Hat1^−/−^ mouse embryonic fibroblasts (MEFs) are defective in cell proliferation and are sensitive to DNA damaging agents. In addition, the Hat1^−/−^ MEFs display a marked increase in genome instability. Analysis of histone dynamics at sites of replication-coupled chromatin assembly demonstrates that Hat1 is not only responsible for the acetylation of newly synthesized histone H4 but is also required to maintain the acetylation of histone H3 on lysines 9, 18, and 27 during replication-coupled chromatin assembly.

## Author Summary

The packaging of genomic DNA during replication is a highly orchestrated process. An important aspect of chromatin assembly is the processing of newly synthesized histones prior to their incorporation into chromatin. The transient acetylation of histone H3 and H4 NH_2_-terminal tails is a hallmark of this processing with newly synthesized molecules of histone H4 being predominantly diacetylated. This diacetylation occurs specifically on lysine residues 5 and 12 and this precise pattern is widely conserved throughout eukaryotic evolution. The acetylation of newly synthesized histones is catalyzed by type B histone acetyltransferases. Hat1 is the founding member of this class of enzymes and has been proposed to be responsible for the diacetylation of newly synthesized histone H4. Here we describe the development of a mouse knockout model of Hat1. The absence of Hat1 results in neonatal lethality due to developmental defects in the lung. Mouse embryonic fibroblasts derived from Hat1^−/−^ mice are sensitive to DNA damaging agents and display a high level of genome instability. Biochemical analyses provide definitive evidence that Hat1 is the sole enzyme responsible for the acetylation of newly synthesized histone H4. Surprisingly, Hat1 is also necessary for the normal processing of newly synthesized histone H3.

## Introduction

The packaging of genomic DNA during replication is a highly orchestrated process that ensures both the necessary compaction of the DNA and the proper transmission of the epigenetic landscape [1], [2], [3], [4], [5]. An important aspect of chromatin assembly is the processing of newly synthesized histones for their incorporation into chromatin. The transient acetylation of histone H3 and H4 NH_2_-terminal tails is a hallmark of this processing. Newly synthesized molecules of histone H4 are predominantly diacetylated. This diacetylation occurs specifically on lysine residues 5 and 12 and this precise pattern is widely conserved throughout eukaryotic evolution. The acetylation of histone H3 occurs on a smaller fraction of the newly synthesized molecules and does not occur in a consistent pattern across eukaryotes. A role for this acetylation in histone deposition was first suggested by the correlation between the presence of these histone marks and active chromatin assembly as H3 and H4 are rapidly modified after their synthesis and then deacetylated following their incorporation into chromatin [6]. However, despite this longstanding correlation, an understanding of the function of histone NH_2_-terminal tail domain acetylation in chromatin assembly remains elusive.

In addition to their NH_2_-terminal tail domains, evidence from *S. cerevisiae* indicates that newly synthesized histones are also acetylated in their core domains with H3 acetylated on lysine 56 and H4 acetylated on lysine 91 [7], [8], [9], [10]. H3 lysine 56 lies near the entry/exit point of the nucleosome in close proximity to the DNA. The acetylation of this site occurs specifically in S phase and has been linked to chromatin assembly by a number of observations. First, mutations in yeast that alter H3 lysine 56 cause defects in the reassembly of chromatin structure that accompanies the recombinational repair of a DNA double strand break. Second, H3 lysine 56 mutations influence the binding of histone H3 to the CAF-1 histone chaperone complex that plays a key role in replication coupled chromatin assembly [7], [11], [12], [13], [14], [15], [16], [17]. Histone H4 lysine 91 lies in the interface between H3/H4 tetramers and H2A/H2B dimers where it forms a salt bridge with an aspartic acid residue in histone H2B. Hence, the acetylation of H4 lysine 91 may regulate tetramer-dimer interactions and genetic results are consistent with a role for this modification in chromatin assembly [10], [18].

Enzymes known as type B histone acetyltransferases (HATs) catalyze the acetylation of newly synthesized histones. Type B HATs are primarily distinguished from type A HATs by their substrate specificity. As expected for enzymes that modify histones prior to their assembly into chromatin, type B HATs are highly specific for free histones. Type B HATs may also function outside of the nucleus [19]. A number of type B HATs have now been identified. The first was Hat1p, which acetylates free histone H4 on lysine residues 5 and 12 [20], [21]. In addition, the yeast enzyme Rtt109p acetylates free histone H3 on lysine 56 and lysine 9 [22], [23], [24]. Interestingly, the original type A HAT, Gcn5p, may also possess type B HAT activity in *S. cerevisiae* as it has been shown to be involved in the acetylation of the NH_2_-terminal tail of newly synthesized histone H3 [25], [26]. Finally, the mammalian enzyme HAT4 may also be a type B HAT as it resides in the Golgi and is capable of acetylating histone H4 lysine 91 [27].

Originally isolated from budding yeast, Hat1p was found to exist in at least 2 complexes. The first is a cytoplasmic complex that also contains Hat2p, which is a homolog of the Rbap46 histone chaperone in mammalian cells [21], [28]. Hat1p is also found in a nuclear complex that, in addition to Hat2p, contains another histone chaperone, Hif1p (a homolog of the mammalian protein NASP) [29]. Not only the Hat1p protein itself but also the Hat1p-containing complexes are highly conserved in eukaryotes. Complexes with similar compositions have been isolated from human, frog, chicken and corn. As expected from their high degree of similarity, these enzymes specifically acetylate free histone H4 on lysines 5 and 12 [30], [31], [32], [33].

Despite its widespread conservation, initial genetic analyses in yeast showed that loss of Hat1p had no detrimental effects on either chromatin assembly or cell viability [20], [21], [34]. This lack of phenotypic effect was, at least partly, due to functional redundancy as combining the deletion of *HAT1* with mutations in specific sets of lysine residues in the histone H3 NH_2_-terminal tail resulted in defects in telomeric silencing and DNA damage sensitivity [35], [36]. DNA damage sensitivity has also been observed in *S. pombe* and chicken DT40 cells lacking Hat1 [37], [38]. Importantly, direct evidence linking yeast Hat1p to chromatin assembly in the contexts of DNA damage repair and histone exchange has recently been reported [39], [40].

Despite the minor effects on cell viability observed in the absence of Hat1, biochemical analyses have implied that Hat1 may play a critical role in histone processing and dynamics. This is suggested by an intriguing property of Hat1p. Unlike most enzymes, Hat1 appears to remain stably associated with its histone substrates following acetylation [41]. This property of Hat1p also appears to be widely conserved. In yeast, both the cytoplasmic and nuclear Hat1p-containing complexes are stably associated with histones H3 and H4 [29]. In mammalian cells, Hat1p appears to be one of the primary proteins physically associated with soluble histones [42], [43], [44], [45], [46], [47]. Therefore, Hat1p has the potential to function both catalytically and stoichiometrically in the chromatin assembly process.

To explore the function of Hat1 and the acetylation of newly synthesized histones in mammals, we have generated a conditional Hat1 knockout mouse model. Hat1^−/−^ animals are neonatal lethal with developmental lung defects. These result from hyper proliferation of mesenchymal cells leading to severe atelectasis, less aeration and death upon respiratory failure. In addition, a significant fraction of the Hat1^−/−^ animals display severe craniofacial defects. Mouse embryonic fibroblasts (MEFs) derived from Hat1^−/−^ embryos show multiple defects including slow growth, DNA damage sensitivity and genome instability. Analysis of proteins present on newly replicated DNA by iPond (isolation of proteins on nascent DNA) indicates that histones H3 and H4 deposited during replication-coupled chromatin assembly are hypo-acetylated in the absence of Hat1 [48]. Consistent with these observations, analysis of newly synthesized histones indicates that Hat1 is the sole HAT responsible for the acetylation of newly synthesized histone H4. Surprisingly, loss of Hat1 also leads to a decrease in the modification of newly synthesized histone H3. These results demonstrate that Hat1 is essential in mammals and that it plays an integral role in the processing of newly synthesized histones during the process of chromatin assembly.

## Results

### Generation of a conditional Hat1 knockout mouse model

**Figure 1:**
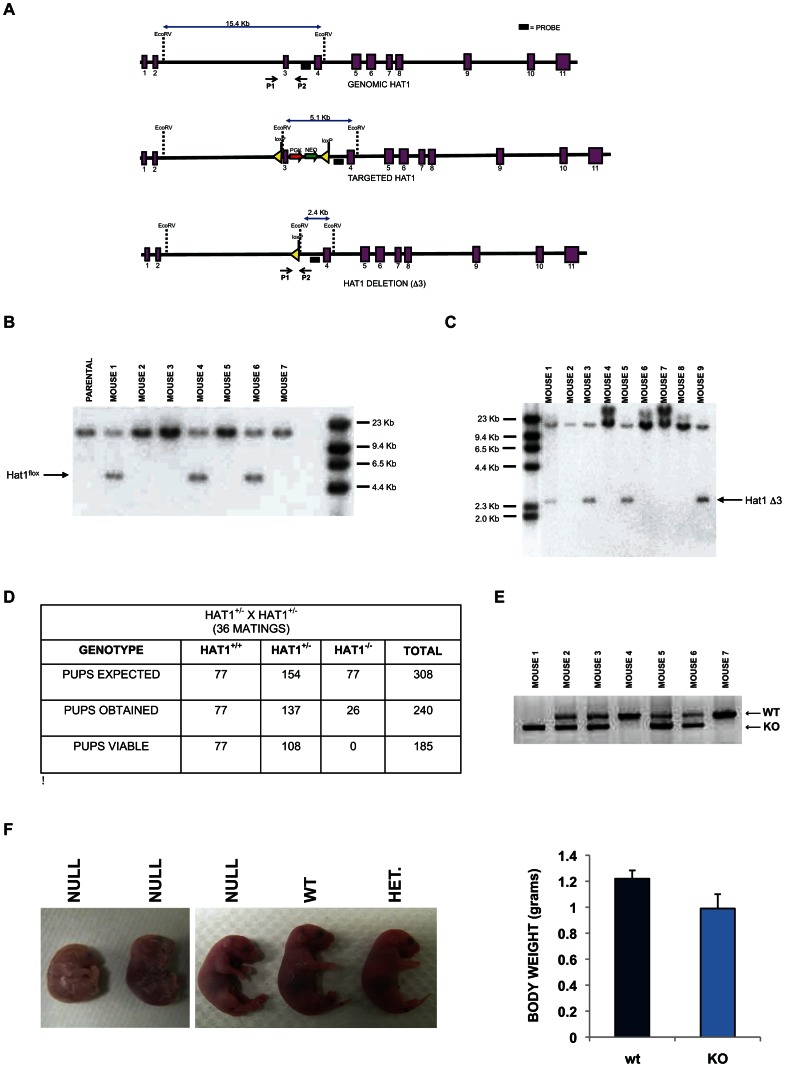
Histone acetyltransferase 1 is essential for viability in mice. A) Schematic diagram of the wild type mouse Hat1 locus (top), the Hat1 locus following integration of loxP sequences flanking intron three (middle) and the Hat1 locus following Cre mediated deletion of exon 3. Exons are represented by purple rectangles. Locations of probes and PCR primers are indicated. (B) Genomic DNA isolated from a parental (C57/bl6) mouse and mice generated from a cross between a chimeric mouse and a wild type mouse was digested with EcoRV and analyzed by Southern blot using the indicated probe. Mice 1, 4 and 6 are Hat1^flox/WT^. C) A Hat1^flox/WT^ mouse was crossed with a mouse that ubiquitously expresses the cre recombinase. Genomic DNA was isolated from mice generated by this cross, digested with EcoRV and analyzed by Southern blot with the indicated probe. Mice 3, 5 and 9 are WT/KO/Cre. D) Table lists the number of expected, obtained and viable pups of the indicated genomes derived from matings of Hat1^+/−^ mice. E) PCR genotyping of a representative litter from a Hat1^+/−^ X Hat1^+/−^ mating. Primers P1 and P2 (see above) were used for amplification. Arrows indicate specific PCR products. F) Representative neonatal pups from Hat1^+/−^ X Hat1^+/−^ matings with the indicated genotype. F) Body weight of pups was measured immediately following birth. Data are derived from 10 pups of each genotype.

There is a single homolog of Hat1 in the murine genome that is highly similar to human and yeast Hat1. The murine Hat1 gene consists of 11 exons (Figure 1A). A construct was generated to target the integration of loxP sites to flank Hat1 exon 3. In the presence of cre recombinase, exon 3 can be deleted with the subsequent introduction of a stop codon (Figure 1A). This will create a truncation mutant of Hat1 that produces a product less 50 amino acids long. In the event that alternate splicing occurs in the Hat1 gene that could skip exon 3, only exon 9 can be spliced to exon 2 and retain the proper reading frame. In this case, the protein that would be produced would lack the entire Hat1 active site.

The targeting construct was transfected into mouse embryonic stem (ES) cells and cells grown with antibiotic selection. Cell lines in which the targeting construct was properly integrated were identified (data not shown). These cells were then injected into blastocysts to generate chimeric mice. The chimeras were then mated with wild type mice (C57/Bl6) and the pups were screened by Southern blot to determine whether germline transmission of the Hat1^flox^ allele had been achieved. Several animals with a Hat1^flox^/Hat1^+^ genotype were identified (Figure 1B).

To generate a complete Hat1 mouse knockout, the Hat1^flox^/Hat1^+^ mice were mated to mice that ubiquitously express the Cre recombinase. The litters from these matings were screened to identify offspring in which Hat1 exon 3 had been deleted (Hat1^Δ3^). As seen in Figure 1C, several mice were obtained with the genotype Hat1^Δ3^/Hat1^+^. These mice also carried a copy of the Cre recombinase gene. We backcrossed these mice with C57/Bl6 mice to obtain animals that no longer expressed Cre to avoid any undesired phenotypic consequences that could arise from expression of this recombinase. Following backcrossing to remove cre from the genome, Hat1^+^/Hat1^Δ3^ mice were mated and the genotypes of the resulting pups were determined (Figure 1D,E). For simplicity, Hat1^Δ3^ mice will be referred to as Hat1^−^. As seen in Figure 1D, based on the number of Hat1^+/+^ pups born, there were slightly fewer than expected Hat1^+/−^ pups and a marked decrease in the number of Hat1^−/−^ pups born. Importantly, all of the Hat1^−/−^ pups were either born dead or died shortly after birth (Figure 1F). In addition, the Hat1^−/−^ pups were approximately 20% smaller than their Hat1^+/+^ counterparts (Figure 1F).

### Hat1 is necessary for proper mammalian development

**Figure 2:**
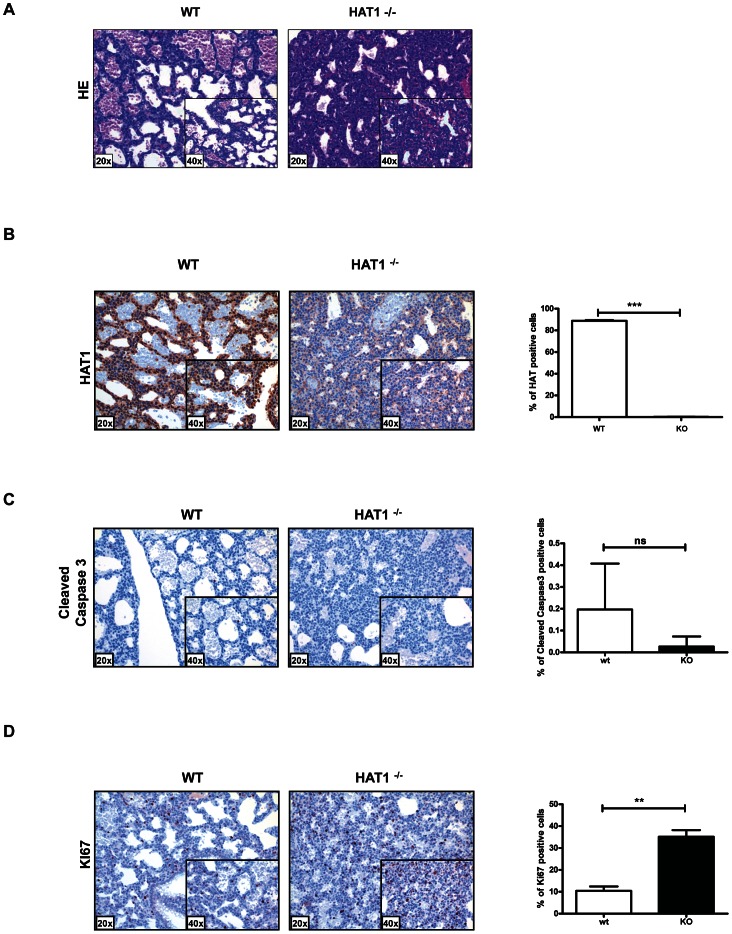
Developmental lung defects result in neonatal death in the absence of Hat1. A) Histologic appearance of lungs from newborn pups obtained from a Hat1^+/−+^ and Hat1^−/−^ mice. Staining was with hematoxylin-eosin; magnification 20×, (inlets ×40). The lungs of Hat1^−/−^ show less aeration, due to thickened mesenchyme resulting in death due to respiratory failure. B) Hat1 is highly expressed in lungs of Hat1^+/+^ but not in Hat1^−/−^ mice; magnification 20×, (inlets ×40) C) Cleaved Caspase3 stained by IHC showed no difference between lungs of Hat1^++^ and Hat1^−/−^ mice; magnification 20×, (inlets ×40) D) Ki67 stained by IHC shows significantly higher proliferation rates in lungs of Hat1^−/−^ mice compared to controls; magnification 20×, (inlets ×40). Quantification was done by HistoQuest software.

Contrary to what is observed in the other model organisms that have been examined, Hat1 is necessary for viability in mice as the loss of this enzyme results in neonatal lethality. To determine the cause of this lethality, Hat1^+/+^ and Hat1^−/−^ neonates were subjected to pathological examination. Significantly more cells per alveolar septum, which is a measure of fetal lung immaturity, were observed in the Hat1^−/−^ neonates compared to the WT mouse lung (Figure 2A). The lungs from the neonatal Hat1^−/−^ pups also showed a lower overall lung maturation, which was compiled by an assessment of vascularity, aerated lung tissue and septum thickness. These defects in lung development resulted in atelectasis, less aeration and finally lead to respiratory failure (Figure 2A). Lungs of Hat1^+/+^ controls were completely normal (Figure 2A). Hat1 is highly expressed in alveolar as well as lung interstitial cells of Hat1^+/+^ mice (Figure 2B). A highly significant increase in Ki67+ proliferation rates was observed in Hat1^−^/^−^ compared to Hat1^+/+^ neonates. However cleaved Caspases 3 expression was not altered suggesting that the developmental defect was the result of inappropriate proliferation rather than a defect in apoptosis (Figure 2C,D). The inappropriate proliferation begins early in development and is apparent by 11.5 d.p.c. (Figure S1).

**Figure 3:**
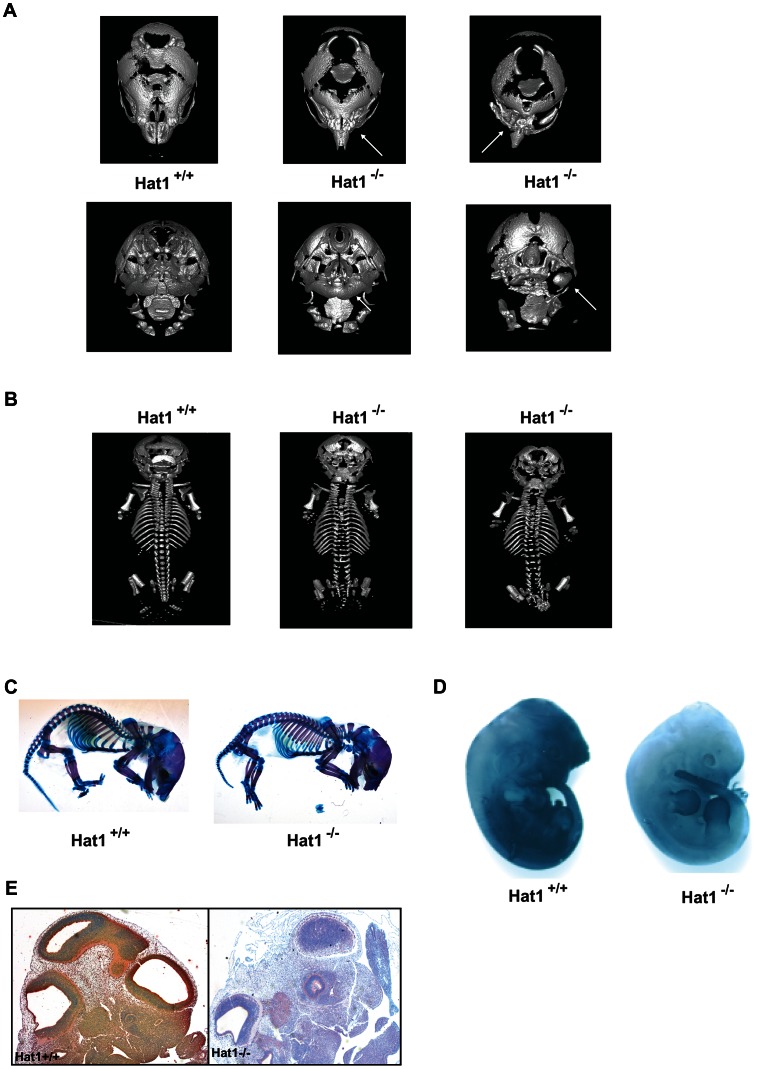
Skeletal defects associated with the loss of Hat1. A) Micro CT scans of the heads of neonates with the indicated genotype. Top row shows a dorsal view and bottom row shows a ventral view. Arrows indicate defects in the nasal cavity (top) and jaw structures (bottom). B) Micro CT scans of Hat1^+/+^ and Hat1^−/−^ neonates (as indicated) showing a dorsal view of the entire animal. C) Acian blue and Alizarin red stained Hat1^+/+^ and Hat1^−/−^ neonates. D) Hat1^+/+^ and Hat1^−/−^ embryos (12.5 dpc) were stained with α-Hat1 antibodies. E) Cross section of the head and neck of 11.5 dpc Hat1^+/+^ and (WT) and Hat1^−/−^ embryos stained with α-Hat1 antibody. 2.5× magnification.

There was another more obvious, but less penetrant, phenotype observed in the Hat1^−/−^ mice. Approximately 25% of the Hat1^−/−^ neonates were born with craniofacial abnormalities. The skeletal structures of Hat1^+/+^ and Hat1^−/−^ neonates were examined by microCT scanning. As seen in Figure 3A, the structure of the skulls from a number of the neonates is highly abnormal. For example, the nasal passages were often missing, having been overgrown with bone. In addition, there were severe defects in the development of the lower jaw structure. These defects included several neonates where the lower jaw was missing in its entirety (Figure 3A, right-hand panels) or examples where the lower jaw bones were fused into a single bone (Figure 3A, middle panel). Defects to the remainder of the skeletal system were less severe. As shown in Figure 3B, while the upper areas of the spine are relatively normal, the structure of the vertebrae in the Hat1^−/−^ neonates degenerate near the base of the spinal column.

To characterize the skeletal defects in more detail, WT and Hat1^−/−^ neonates were stained with Alcian blue and Alizarin red. Alcian blue stains cartilage blue and Alizarin red stains bone purple. Consistent with the microCT results, bone and cartilage staining is similar for the majority of the skeletal system in the WT and Hat1^−/−^ neonates (Figure 3C). However, there are differences in the head. The Hat1^−/−^ neonates showed a marked increase in bone density in the skull and a decrease in the amount of cartilage staining. These results are consistent with the defects seen in lung development as the increased bone density may be the result of osteoblast hyper-proliferation in the absence of Hat1.

To correlate embryonic expression of Hat1 with the skeletal defects observed in the Hat1^−/−^ neonates, whole embryos were stained with α-Hat1 antibodies. As seen in Figure 3D, there is widespread expression of Hat1 protein in the head of embryos. There is also a high level of staining in the abdominal region. Closer examination of Hat1 in the head by immunohistochemistry showed that Hat1 is widely expressed in most tissue types (Figure 3E). Therefore, the phenotypes observed in the Hat1^−/−^ neonates are not strictly linked sites of Hat1 protein expression.

### Hat1 is necessary for DNA damage repair and genome stability

**Figure 4:**
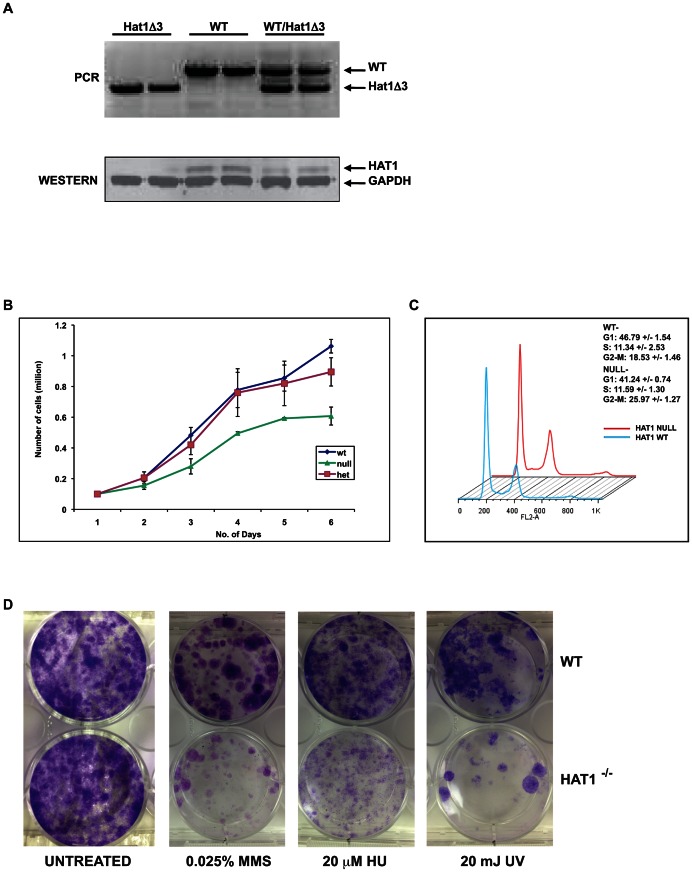
Hat1^−/−^ MEFs display cell proliferation and DNA damage repair defects. A) Hat1^+/+^, Hat1^+/−^ and Hat1^−/−^ primary MEFs were genotyped by PCR as described in the legend to Figure 1. Whole cell extracts from the indicated MEFs were analyzed by Western blots probed with the indicated antibodies. B) Equal numbers of primary MEFs of the indicated genotype were seeded at time zero. Cell numbers were counted at the indicated time points. C) Primary Hat1^+/+^ and Hat1^−/−^ MEFs were stained with propidium iodide and analyzed by FACS. Fraction of cells in each phase of the cell cycle is indicated. D) Immortalized Hat1^+/+^ and Hat1^−/−^ MEFs were grown under the indicated conditions. Plates were photographed after crystal violet staining.

The fact that Hat1^−/−^ offspring survive to at least late embryogenesis facilitated the generation of Hat1^−/−^ embryonic fibroblast cell lines to address specific questions about the function of Hat1 in mammalian cells. Mouse embryonic fibroblasts (MEFs) were generated from Hat1^+/+^, Hat1^+/−^ and Hat1^−/−^ embryos (Figure 4A). Western blot analysis using α-Hat1 antibodies confirmed that the MEFs isolated from the Hat1^−/−^ embryos were completely devoid of Hat1 protein (Figure 4A). In addition, heterozygous MEFs (isolated from Hat1^+^/Hat1^−^ embryos) showed an ∼2-fold decrease in Hat1 protein levels.

The loss of Hat1 protein does not influence cell proliferation in any of the model organisms in which it has been genetically deleted (yeast and avian cells). To determine whether Hat1 was important for mammalian cell proliferation, growth curves were measured for the WT, heterozygous and Hat1 null MEFs. As seen in Figure 4B, only minor differences in proliferation between the WT and heterozygous cells were observed. However, Hat1 null cells showed an ∼50% decrease in cell proliferation.

To determine whether the decrease in cell proliferation seen with the Hat1^−/−^ MEFs was the result of a specific defect in cell cycle progression, FACS analysis was used to monitor cell cycle distribution. As seen in Figure 4C, Hat1^−/−^ MEFs displayed a moderate accumulation of cells in G2/M suggesting that the decrease in cell proliferation seen in the absence of Hat1 may be, at least in part, due to a G2/M delay in these cells. Taken together, these results indicate that Hat1 protein is not essential for the proliferation of mammalian cells but that cell cycle progression is defective in the absence of this enzyme.

Loss of Hat1 in budding yeast, fission yeast and chicken DT40 cells results in the sensitivity to DNA damaging agents [36], [37], [38]. To determine whether a role for Hat1 in DNA damage repair is conserved in mammalian cells, WT and Hat1^−/−^ MEFs were assayed for their sensitivity to a variety of DNA damaging agents. To avoid complications arising from the limited proliferation potential of primary MEFs, immortalized cells lines from Hat1^+/+^ and Hat1^−/−^ primary MEFs were generated via transfection with SV40 large T antigen (proliferation rates of immortalized MEFs are shown in Figure S2). Equal numbers of Hat1 WT and Hat1 null cells were plated and allowed to grow in normal serum containing either methyl methane sulfonate (MMS) or hydroxyurea (HU) or following exposure to ultraviolet radiation (UV) (Figure 4D). The Hat1^−/−^ cells showed a pronounced sensitivity to each of these DNA damaging agents. Hence, Hat1 plays a critical role in DNA damage repair in mammalian cells. Interestingly, the Hat1^−/−^ mammalian cells were sensitive to a broader range of DNA damaging agents. Both yeast and avian cells lacking Hat1 are sensitive to MMS but not UV radiation, suggesting that these Hat1^−/−^ mutants are specifically sensitive to DNA double strand breaks [36], [37], [38]. However, the Hat1^−/−^ MEF cell lines sensitivity to both types of DNA damage indicating that Hat1 is important for multiple pathways of DNA repair.

**Figure 5:**
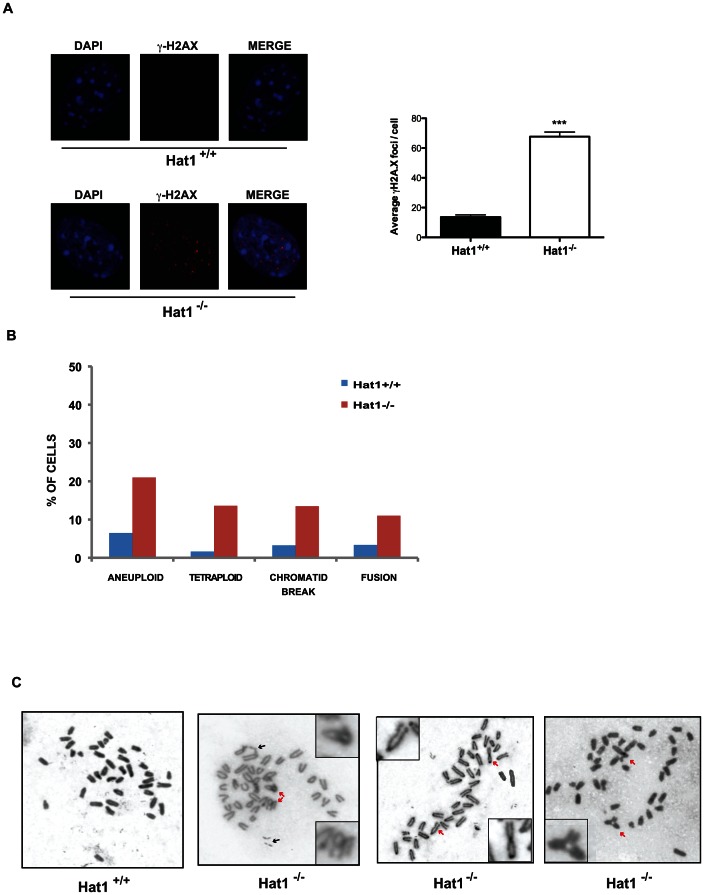
Hat1 is essential for maintaining genome stability. A) Hat1^+/+^ and Hat1^−/−^ MEFs were stained with either DAPI or α-γH2AX antibodies as indicated (left). Visible γH2AX foci were counted in 12 cells of each genotype (right). B) Metaphase spreads from Hat1^+/+^ and Hat1^−/−^ MEFs were analyzed for chromosome number and the presence of breaks and fusions. The percentage of cells containing the indicated chromosomal abnormality is given. The number of spreads analyzed was 56 (Hat1^+/+^) and 112 (Hat1^−/−^). C) Metaphase spreads were generated from Hat1^+/+^ and Hat1^−/−^ MEFs. Insets show enlarged views of selected abnormal chromosomes. Red arrows indicate examples of chromosome fusions and blue arrows indicate chromosome breaks.

As loss of Hat1 resulted in sensitivity to DNA damage, we next explored whether Hat1 was also necessary for proper genome stability. One hallmark of genome instability is the presence of DNA damage in the absence of treatment with DNA damaging agents. Hat1^+/+^ cells showed undetectable levels of endogenous DNA damage, as measured by the presence of γ-H2AX foci (Figure 5A). In contrast, untreated Hat1^−/−^ cells showed numerous γ-H2AX foci. An increase in γ-H2AX levels, both before and after DNA damage, in Hat1^−/−^ MEFs was confirmed by Western blot analysis of whole cell extracts (Figure S3).

To directly visualize genome structure, we generated metaphase spreads from Hat1 WT and Hat1 null cells. Genomic abnormalities that were more frequently observed in the Hat1^−/−^ cells than in the Hat1^+/+^ cells were of several different types (Figure 5B). First, there was a significant increase in chromatid breaks and chromosome fusions. Representative examples are shown in Figure 5C (additional metaphase spreads are shown in Supplemental Figure S4). In this figure, black arrows indicate examples of chromatid breaks or gaps. Red arrows indicate examples of chromosomal fusions. These included both “end-to-end” fusions and unusual “bridge-like” structures where the ends of one chromosome were fused to internal regions of another chromosome. We also observed changes in chromosome number. There was an increase in the number of aneuploid cells that contained a smaller than normal number of chromosomes in the Hat1^−/−^ cells. In addition, there were high numbers of Hat1^−/−^ cells with a 4n DNA content (Figure 5C). In summary, the absence of Hat1 resulted in the presence of high levels of endogenous DNA damage and chromosomal abnormalities, indicating that Hat1 plays an essential role in maintaining genome stability.

### Hat1 is essential for the processing of histones H3 and H4 during replication-coupled chromatin assembly

Since its initial discovery and biochemical characterization, Hat1 has been presumed to be involved replication-coupled chromatin assembly through the conserved diacetylation of newly synthesized histone H4. However, evidence to support this hypothesis has been circumstantial [41], [49]. The availability of mammalian cells genetically deleted for Hat1 allowed us to definitively address this issue. To directly determine whether Hat1 is involved in the acetylation of histones that are incorporated during replication coupled chromatin assembly, we used iPond to monitor histone modification dynamics on newly replicated DNA [48]. The iPond technique involves pulse-labeling cells with the thymidine analog EdU. The EdU will then be incorporated into DNA that is synthesized during the pulse phase. Following cross-linking, Click chemistry can then be used to covalently attach biotin to the EdU moieties, which allows for the affinity purification of the nascent DNA using streptavidin beads. Western blot analysis of the fractions that elute from the streptavidin beads can then be used to monitor the presence of specific proteins or their modified isoforms on the newly synthesized DNA.

**Figure 6:**
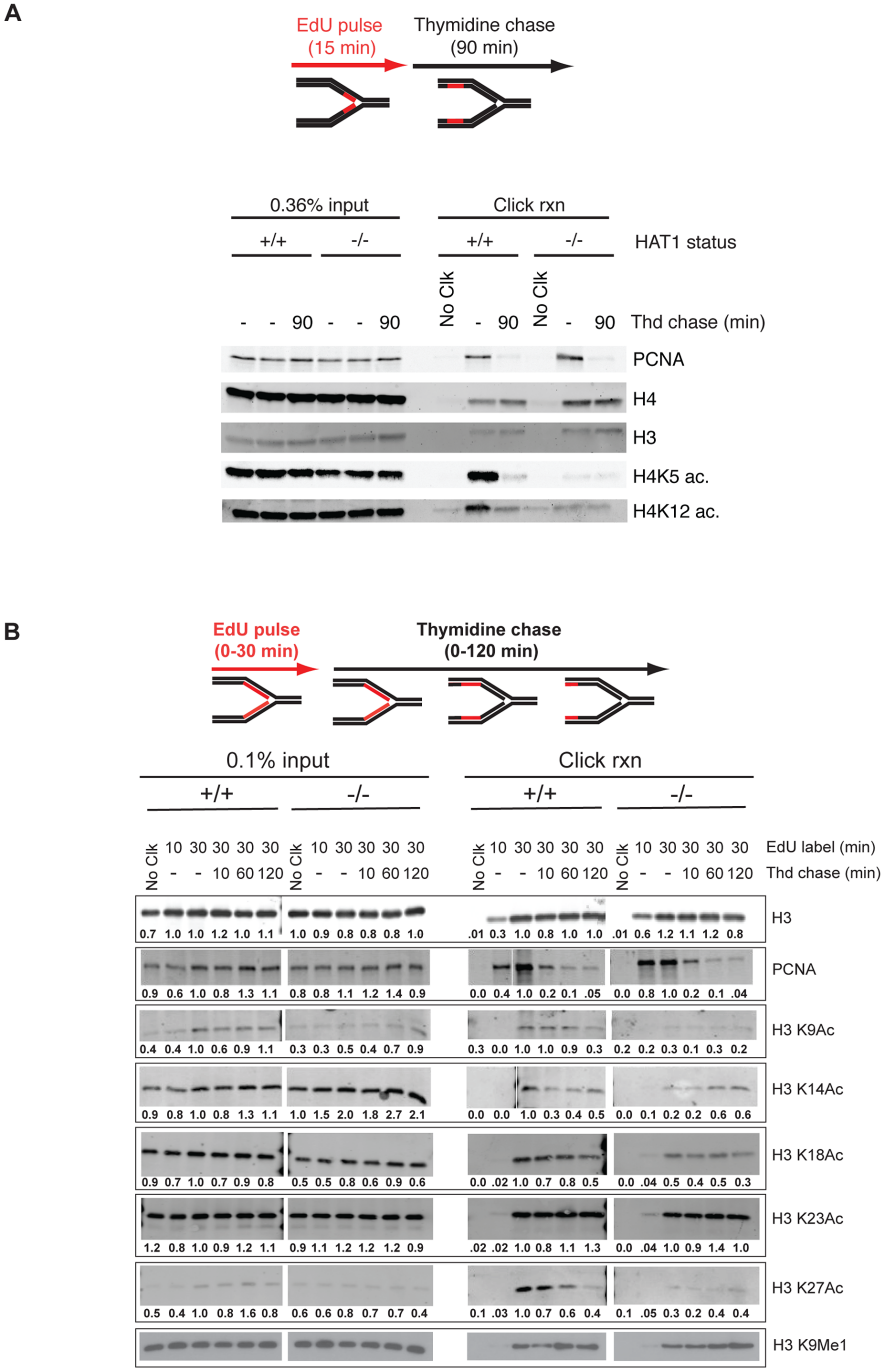
Hat1 is required for the acetylation of histones H3 and H4 deposited during replication-coupled chromatin assembly. A and B) Hat1^+/+^ and Hat1^−/−^ MEFs were pulse-labeled with EdU and chased with thymidine as schematically depicted at the top of each panel. Samples were isolated at the indicated time points and proteins associated with nascent DNA were resolved by SDS-PAGE following affinity purification of EdU-labeled DNA (iPond). The indicated amounts of the input fractions (prior to affinity purification) are on the left-hand side of each panel. Samples eluted from the affinity purification resin are on the right-hand side of each panel. In all cases, the respective Hat1^+/+^ and Hat1^−/−^ samples were analyzed on the same gel (separated by a MW standard lane that has been removed). Western blots were probed with the antibodies indicated on the right. Numbers below each lane indicate the normalized intensity of the band as determined using Licor software. Each sample was normalized to the level of unmodified histone H3. The intensity observed in the 30′ pulse sample was arbitrarily set to 1.0. No Clck indicates control samples that were not biotin labeled.

Immortalized Hat1^+/+^ and Hat1^−/−^ MEFs were pulsed with EdU for 15 minutes and then chased with thymidine for 90 minutes. The 90 minute thymidine chase allowed us to distinguish between stably associated chromatin proteins and proteins that associate with newly replicated DNA but then are removed from chromatin after replication. For example, the DNA replication factor PCNA is found associated with nascent DNA immediately following the EdU pulse but is largely absent following the 90 minute thymidine chase while the levels of histone H3 and H4 remain constant (Figure 6A, right panel). It is important to note that the levels of PCNA and histones H3 and H4 do not vary between the Hat1^+/+^ and Hat1^−/−^ MEFs indicating that the rate of EdU incorporation is not altered by the loss of Hat1. As previously reported, the levels of histone H4 lysine 5 and lysine 12 acetylation are high on nascent DNA and then decrease over the 90 minute thymidine chase in the Hat1^+/+^ MEFs [48]. In the Hat1^−/−^ cells there is a striking decrease in the levels of H4 lysine 5 and 12 acetylation on the nascent DNA. The low level of acetylation that remains does not decay over time and is consistent with the observation that parental histones can remain acetylated during their reassembly during DNA replication [50]. These results indicate that Hat1 is likely to be the sole histone acetyltransferase involved in the acetylation of histone H4 lysines 5 and 12 during replication coupled chromatin assembly.

We also examined the dynamics of histone H3 modification during replication-coupled chromatin assembly. In these experiments cells were given either 10 or 30 minute pulses of EdU. Following the 30-minute EdU pulse, the cells were given a thymidine chase for 10, 60 or 120 minutes. Surprisingly, examination of the input samples indicated that loss of Hat1 impacted the steady state levels of acetylation at specific H3 lysine residues (Figure 6B, left panel). When normalized to unmodified histone H3, there was a marked decrease in the overall abundance of acetylated H3 lysine 9 (>2-fold)and a moderate decrease in the acetylation of lysines 18 and 27 (<2-fold). In addition, there was an increase in the level of H3 lysine 14 acetylation (∼2-fold).

Importantly, Hat1 also had a significant influence on the dynamics of histone H3 acetylation on nascent DNA. In both Hat1^+/+^ and Hat1^−/−^ cells, total histone H3 levels increased during the pulse, as more nascent DNA was labeled, and then remained constant throughout the chase (Figure 6B, right panel). As expected, PCNA levels increased during the EdU pulseand then decreased during the thymidine chase [48]. Interestingly, distinct patterns of acetylation dynamics were observed for subsets of the lysine residues on the NH_2_-terminal tail of histone H3. The acetylation of H3 lysines 14 and 23 largely mirrored that of bulk H3 where the levels remained relatively stable during the thymidine chase. This acetylation was not significantly influenced by the loss of Hat1. A second pattern, seen for lysines 9, 18 and 27, displayed kinetics similar to those seen for H4 lysine 5 and 12 acetylation where acetylation increased during the EdU pulse and then decayed to a basal level during the thymidine chase. Surprisingly, the acetylation of lysines 9, 18 and 27 was sensitive to the loss of Hat1 and showed only a basal level of acetylation that did not decay during chromatin maturation. Therefore, in addition to its expected effects on histone H4, the presence of Hat1 is also essential for acetylation of histone H3 deposited during replication-coupled chromatin assembly.

In addition to acetylation, newly synthesized histone H3 is also monomethylated on lysine 9. In fact, H3 lysine 9 monomethylation appears to be precede any other modifications on H3 and H4 [43], [51]. H3 lysine 9 monomethylation was apparent on nascent DNA and then increased during the course of the thymidine chase (Figure 6B). The levels and kinetics of H3 lysine 9 monomethylation were not influenced by the absence of Hat1. Hence, the mono-methylation of newly synthesized histone H3, which is thought to occur prior to its association with the Hat1 complex, is not dependent on Hat1.

### Hat1 is essential for the acetylation of newly synthesized histones

**Figure 7:**
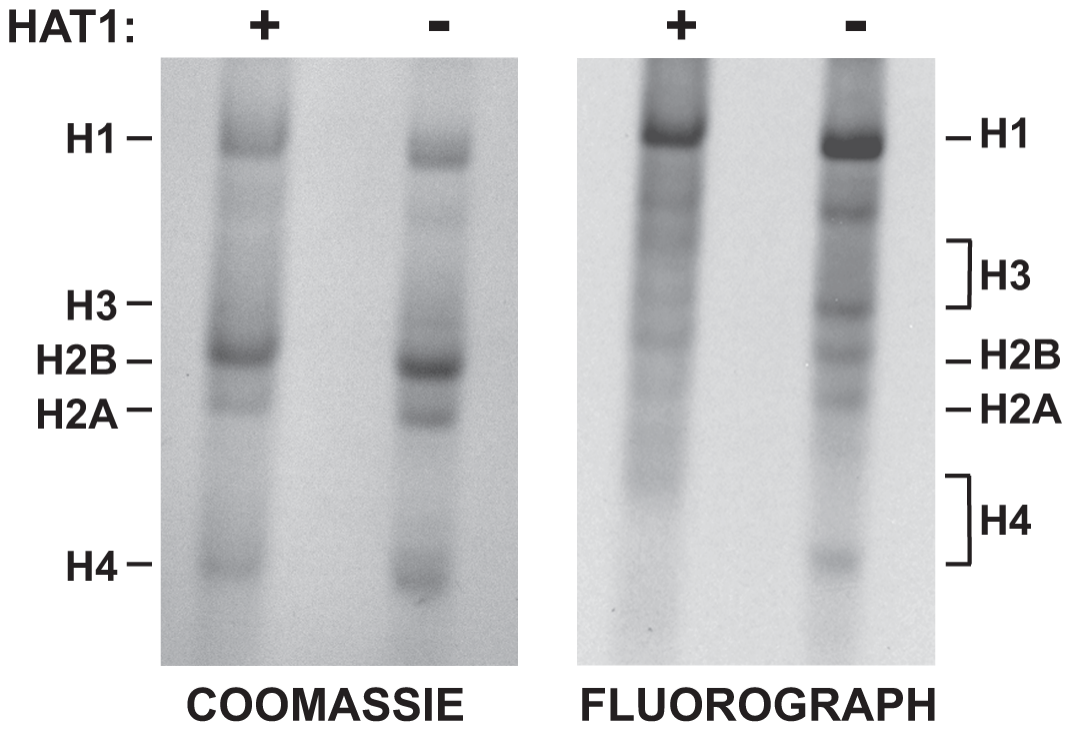
Hat1 is essential for the acetylation of newly synthesized histones. Hat1^+/+^ and Hat1^−/−^ MEFs were pulse-labeled with ^3^H-lysine for 12 minutes. Histones were then isolated and resolved by Acid-Urea (AU) gel electrophoresis. Total protein was visualized with Coomassie blue staining and radio-labeled proteins visualized by fluorography (as indicated). The mobility of each histone is indicated. The brackets indicate the regions of mobility for the acetylated isoforms of histone H4 and histone H3.

The effect of Hat1 on the acetylation state of histones incorporated during replication-coupled chromatin assembly suggested that Hat1 is modifying newly synthesized molecules. To test this, Hat1^+/+^ and Hat1^−/−^ MEFs were briefly pulsed with ^3^H-lysine to radiolabel newly synthesized proteins. Histones were then purified from these cells by acid extraction and resolved by acid-urea (AU) gel electrophoresis. AU gels are capable of resolving the acetylated isoforms of histones where the addition of each acetyl groups causes a successive decrease in electrophoretic mobility. The AU gels were stained with coomassie blue and then processed for fluorography (Figure 7). The coomassie blue stained gel shows the mobility and distribution of bulk histones. The absence of Hat1 had little effect on the bulk histones. Examining the radiolabeled histones provides specific information on the distribution of acetylated isoforms of the newly synthesized histones. In Hat1 WT cells, essentially all of the newly synthesized histone H4 migrated at a position consistent with the diacetylated state, in agreement with previous reports. However, in the absence of Hat1, it appeared that nearly all of the newly synthesized histone H4 was found to be unacetylated. This conclusively demonstrates that Hat1 is involved in the acetylation of newly synthesized histone H4 and appears to be the only enzyme responsible for this pattern of acetylation.

Surprisingly, loss of Hat1 also altered the distribution of newly synthesized histone H3. While histone H3 is more difficult to resolve in AU gels, newly synthesized histone H3 isolated from Hat1 WT cells showed a distribution of isoforms. In the absence of Hat1, there was a decrease in the modified isoforms and a marked increase in unacetylated newly synthesized histone H3. This is consistent with the effect of Hat1 on the acetylation state of H3 deposited during replication-coupled chromatin assembly and suggests the possibility that the proper processing of newly synthesized histone H3 is linked to processing and acetylation of newly synthesized histone H4.

## Discussion

The results presented here provide definitive evidence for the role of mammalian Hat1 in the diacetylation of newly synthesized histone H4 during replication-coupled chromatin assembly. Surprisingly, Hat1 is also essential for the acetylation state of histone H3 incorporated during replication-coupled chromatin assembly. This processing of newly synthesized histones H3 and H4 may be a critical aspect of the regulation of cell proliferation as the absence Hat1 results in defects in lung and cranio-facial development. Further, the absence of Hat1 causes pronounced defects in DNA damage repair and genome stability. Therefore, type B histone acetyltransferases and the acetylation of newly synthesized histones play a fundamentally important role in mammalian cell growth and development.

### In vivo functions of Hat1

Hat1 function has been studied in a number of model organisms, including *S. cerevisiae*, *S. pombe* and chicken DT40 cells. The absence of Hat1 in these organisms did not have any significant impact on overall cell proliferation [20], [21], [34], [37], [38]. Combined with the absence of a significant phenotype arising from mutating histone H4 lysine 5 and 12 in budding yeast had led to the idea that the evolutionarily conserved diacetylation pattern on newly synthesized histone H4 is either not involved in chromatin assembly or plays only an accessory or specialized role in this process [52], [53], [54]. The current analysis of mammalian Hat1 indicates that, while Hat1 is essential for viability in the mouse, it is not essential for cell proliferation. Indeed, the loss of viability seen in the Hat1^−/−^ neonates appears to be the result of specific developmental defects that result in cellular hyperproliferation. It should be stressed that the direct cause of the morphological defects observed in the Hat1^−/−^ neonates is not known. However, potential effects on development due to alterations in chromatin assembly are consistent with the recent report that a mutation in the replication-coupled histone H3 variant, H3.1, or mutations in the histone chaperone CAF-1 cause specific neural development defects in *C. elegans* [55]. Hence, while the current study does not address whether the essential function of Hat1 is related to its impact on histone deposition or through an as yet unidentified cellular role, the use of developmentally complex organisms may facilitate our understanding of the *in vivo* consequences of manipulating chromatin assembly pathways.

A function for Hat1 in DNA damage repair appears to be evolutionarily conserved. However, Hat1 appears to play a more extensive role in mammalian cells than in the other organisms examined. For example, deletion of *HAT1* in S. cerevisiae (when combined with specific mutations in histone H3) results in sensitivity to MMS and to the exogenous expression of restriction endonucleases [36]. Likewise, loss of Hat1 in S. pombe and chicken DT40 cells increases sensitivity to MMS. However, loss of Hat1 in these organisms does not increase sensitivity to UV exposure suggesting that the role of Hat1 is limited to double strand break repair [37], [38]. However, the Hat1^−/−^ MEFs display sensitivity to both double strand and single strand damaging agents. Importantly, mammalian Hat1 mutants also display profound defects in genome instability, which has not been observed elsewhere. These observations suggest that mammalian DNA repair-linked chromatin assembly pathways may be more dependant on the proper modification state of newly synthesized histones or that Hat1 may play a more direct and integral role in DNA repair mechanisms in mammalian cells. In addition, the observation of chromosomal fusions in the absence of Hat1 may reflect a disruption of telomere structure, which is another property of Hat1 mutants that is evolutionarily conserved [56], [57].

The process of chromatin assembly is both spatially and temporally dynamic, which has complicated efforts to definitively demonstrate that Hat1 is involved in histone deposition. The alterations in histone acetylation patterns observed on nascent DNA in Hat1^−/−^ MEFs directly links Hat1 to replication-coupled chromatin assembly. Combined with two other recent reports, the Hat1 enzyme has now been directly linked to the process of chromatin assembly in three distinct contexts. Consistent with the DNA double strand break sensitivity of *hat1*Δ budding yeast, the absence of Hat1 resulted in defects in the reassembly of chromatin structure that is linked to the recombinational repair of DNA double strand breaks [39]. In addition, a genetic screen in yeast identified Hat1 as a factor important for replication-independent chromatin assembly (or histone exchange) [40]. These observations suggest that the acetylation of newly synthesized histones is a ubiquitous feature of all chromatin assembly pathways consistent with the presence of the lysine 5 and 12 diacetylation pattern on both histones H3.1 and H3.3 [43], [50], [51], [58], [59], [60].

While it is clear that Hat1 is involved in chromatin assembly, the precise function of the lysine 5 and 12 diacetylation pattern on newly synthesized histone H4 has not been identified. Recent reports have suggested that this diacetylation pattern promotes the nuclear import of new H4 perhaps through increasing interactions with Importin 4 [42], [51]. However, the analysis of histone dynamics on nascent DNA presented here suggest that in the absence of diacetylation on newly synthesized H4, the level and kinetics of H3 and H4 deposition is similar. This suggests that any impact on nuclear import is not critical for histone deposition during DNA replication in mammalian cells.

### Processing of newly synthesized histone H3

The iPond analysis of the acetylation state of histone H3 during replication-coupled chromatin assembly indicated that histone deposition is accompanied by the transient acetylation of histone H3 lysines 9, 18 and 27. The fact that decay of these acetylations is similar to that of H4 lysine 5 and 12 acetylation and that these acetylations are dependent on Hat1 suggests that this acetylation pattern may be a hallmark of chromatin assembly in murine cells. However, this pattern of acetylation does not match those observed on soluble histones in other mammalian systems. For example, newly synthesized histone H3.1 in HeLa cells appears to be unacetylated while newly synthesized H3.2 and H3.3 showed low levels of acetylation on all of the lysine residues in the H3 NH_2_-terminal tail [50]. In addition, soluble histones H3.1 and H3.3 from HeLa cells showed low levels of acetylation on lysine 9 and moderate levels of acetylation on either lysine 14 or 18 (lysine 27 was not examined) [58], [60]. There may be a number of reasons for this discrepancy. First, acetylation patterns on newly synthesized histone H3 are not conserved across eukaryotes as is the case for histone H4 [61]. Hence, newly synthesized histones in murine cells may be acetylated in a pattern that is different from human tissue culture cells. Second, all of the newly synthesized histones in a cell may not be equivalent. There may be separate pools of soluble histones that have specific modification patterns and these separate pools may be directed to specific chromatin assembly pathways. Finally, soluble histones may not be entirely representative of newly synthesized histones as these pools are likely to contain histones that have been removed from chromatin and, thus, will contain patterns of modification based on their previous location in chromatin.

### Hat1 and the processing of newly synthesized histones

Soluble (and likely cytosolic) histones H3 and H4 are found in multiple discreet complexes [43], [44], [45], [46], [51], [62]. These complexes contain different sets of associated factors and specific post-translational modification patterns on the histones. This has led to the suggestion that newly synthesized histones H3 and H4 are processed in a sequential pathway that ultimately leads to their deposition on nascent DNA [1], [43], [51], [63]. These analyses also suggest that Hat1 plays an early role in this processing pathway. However, a number of steps in the processing of new H3 and H4 appear to occur before its association with Hat1 based on the fact that histone H4 can be found in complexes before it is acetylated on lysines 5 and 12. For example, cytosolic histones H3 and H4 can be found in separate complexes that contain heat shock factors containing heat shock factors (HSC70 and HSP90, respectively). In fact, the first post-translational modifications on new H3 and H4 occur before these complexes are formed as the H3 associated with HSC70 contains monmethylation on lysine 9 and both H3 and H4 contain poly-ADP-ribosylation. Subsequent complexes, which contain H3 and H4 in association with histone chaperones or nuclear import factors, contain histone H4 that is diacetylated on lysines 5 and 12 indicating that association of the newly synthesized histones with Hat1 occurs upstream of these factors [43], [51].

Our data support many aspects of this model. For example, the monomethylation of newly synthesized histone H3 lysine 9 is independent of Hat1 consistent with the occurrence of this modification on H3 molecules prior to their association with Hat1. In addition, acetylation of newly synthesized histone H3, which is likely to occur following nuclear import, is highly dependent on the presence of Hat1. The substrate specificity of Hat1 is highly conserved across a wide range of eukaryotic organisms. Hence, the effect of Hat1 on the acetylation of histone H3 is not likely to be due to the direct acetylation of H3 by Hat1. Rather, Hat1 may be necessary for the integrity of the newly synthesized histone processing pathway [45]. This is supported by our observation that the modification state of newly synthesized histone H3 is altered in the absence of Hat1 (Figure 7). An alternative, and not mutually exclusive, explanation for the apparent impact of Hat1 on the acetylation state of histone H3 during replication-coupled chromatin assembly is that the absence of Hat1 may have a downstream effect on the acetylation of chromatin associated histone H3. This is suggested by the significant decrease in the steady state level of acetylation on H3 lysines 9, 18 and 27 in Hat1^−/−^ cells. Whether it is the presence of Hat1 or the acetylation state of histone H4 that is the key factor in promoting the downstream acetylation of newly synthesized histone H3 will be an interesting question to answer.

## Materials and Methods

### Generation of conditional Hat1 knockout mice

A targeting vector, WT/flox animals and Hat1^+/−^ animals generated using Cre - loxP methodology with support of Ozgene Inc. (Australia). Targeting vector was designed with a 5′ homology arm (6.9 kb), a 3′ homology (6.6 kb), two loxP sites flanking exon 3 and two FRT sites flanking the PGK –neo cassette located downstream of exon 3. All fragments were generated by PCR using 129Sv/J genomic DNA and confirmed with mapping and sequencing to ensure their correct organization. The targeting vector was electroporated in Bruce 4 embryonic stem cells to generate chimeras. Chimeras were then crossed with C57Bl6/J mice to generate Wt/flox animals. To generate global deletion, Wt/flox animals were bred with Ozcre animals expressing cre recombinase to establish heterozygous mice (WT/KO/CRE).

Animals and MEFs were genotyped by either Southern blotting or PCR designed to detect both WT and Hat1Δ3 alleles. Genomic DNA was extracted by standard methods using phenol: chloroform isolation and genotypes were determined by PCR using the following pairs of primers P1: 5′-GCC TGG TGA GAT GGC TTA AAC -3′ and P2: 5′-GCA AGT AGT ATG ACA AGA GGT AGG -3′. PCR was performed under following conditions; 95°C for 50 min followed by 29 cycles at 95°C for 40 sec., 54.6°C for 30 sec. and 72°C for 60 sec. and final extension for 5 min. at 72°C. The WT and mutant alleles yielded product sizes of 916 bp and 478 bp respectively.

All animal use was performed according to the guidelines of The Ohio State University Institutional Animal Care and Use Committee (IACUC) under permit number 2007A0094.

### Generation of primary and immortalized mouse embryonic fibroblasts

e12.5 to e14.5 embryos were dissected from the pregnant female, voided of their internal organs and disaggregated using an 18-gauge syringe. The embryonic tissues were then plated onto 100 mm tissue culture plates and passaged upon confluency. Passage 0 refers to the stage when the embryos were seeded on the plate and every subsequent splitting is referred to as passage 1, 2, 3, etc. These cell lines were cultured and maintained at 37°C using humidified air supplemented with 5% CO_2_ in Dulbecco's modified Eagle medium (DMEM-Sigma) with 15% fetal bovine serum (FBS-Gibco) and 1X Pen/Strep antibiotics (Sigma). Genotypes were confirmed twice by PCR using yolk sac and the resultant cell lines. SV40 T ag immortalized MEFs (iMEFs) were derived from primary WT and Hat1 mutant embryonic day 13.5 embryos. To establish iMEFs, early passage cells were transformed with SV-40 T antigen containing plasmid pBSSVD2005 (ADDGENE, Cambridge, MA). Early passage cells (P>3) were seeded at 25%/well in 6 well plates and transfected with 2 ug of expression vector using Fugene reagent (Roche). Cells were harvested and seeded into 10 cm dishes after 48 hrs of transfection. The cells were split at 1 in 10 dilutions until passage 5.

### Immunohistochemistry

Whole mouse embyro staining was performed by standard procedure. Briefly, Embryos (e.12.5) were fixed in PBS containing 4% Paraformaldehyde (PFA) overnight at 4°C and bleached with 5% H_2_O_2_ in methanol for 4 hr, blocked with PBSMT buffer (3% instant skim milk powder, 0.1% Triton X-100 in PBS) for 2 hr at room temperature and simultaneously incubated with primary antibodies against Hat1 (Abcam, 1/50 dilution) in PBSMT buffer at 4°C over night. After extensive washes in PBSMT buffer for 5 hrs at 4°C followed by incubation with HRP conjugated secondary antibody(1/100 dilution). Finally, embryos were extensively washed in blocking buffer and developed in DAB solution (0.3 mg/ml DBA, 0.5%NiCl_2_). Immunocytochemsitry was performed by standard procedures. Hematoxylin/Eosin and PAS staining were performed with staining kits from DAKO. Slides were also stained with primary antibodies against Hat1 (Abcam, 1/50 dilution), α-Ki67 (Novocastra, NCL-KI67-P) and anti-Cleaved Caspase 3 (Cell Signaling, #9661)Images were captured with a Zeiss AxioImager Z1 microscope. The stainings were quantified with the HistoQuest and TissueQuest software (TissueGnostics GmbH, Vienna, Austria, www.tissuegnostics.com).

### Colony formation assay

Immortalized MEFs (2000/well) were seeded in 6-well plates overnight to adhere and, next day, cells were exposed to either 20 mJ UV radiation, MMS (0.025%) or HU (20 µM). Cells were incubated in complete culture medium for 12 days at 37°C in a humidified 5% C0_2_ chamber. The cells were then rinsed with PBS, fixed in methanol and stained with crystal violet. Following a 20 min. rinse with tap water, the plates were photographed.

### Cytogenetic analysis of metaphase chromosomes

Metaphase chromosomes were prepared from WT and Hat1^−/−^ primary mouse embryonic fibroblasts using standard cytogenetic procedures. Primary MEFs were cultured and treated with 500 ng colcemid for 4 hr to arrest the actively replicating cells in the metaphase stage. The cells were rinsed with PBS and trypsinized to collect the cell pellet. The cell pellet was exposed to hypotonic swelling with 0.056% KCl at 37°C for 15 min. followed by fixation of nuclei with methanol/glacial acetic acid mix (3∶1) and dropping nuclei on pre-warmed slides. The dried slides were stained with Giemsa and mitotic index was visualized with a light microscope. Experiments were performed with two sets of MEFs from each genotype.

### Micro-computed tomography (mCT) analysis

WT and Hat1^−/−^ mCT images were captured using a Siemens Inveon microCT+SPECT (Siemens Preclinical, Knoxville, TN). Each individual image comprised 400 projections/360° at 0.9 degree intervals and was captured with X-ray source energy of 80 KV, 500 mA. Estimated resolution effective pixel size was 19.40 µm. Images were analyzed by using Inveon research workplace version 2.1 software.

### Bone and cartilage staining

WT and Hat1^−/−^ neonatal pups were eviscerated and the skin was removed before fixing in 95% ethanol for 72 hrs. Embryos were then stained in Alcian blue 8GX solution (15 mg Alcian blue, 80 ml 98% ethanol, 20 ml acetic acid) overnight. After a 24 hour rinse in 95% ethanol, they were transferred to 1% KOH for 6 hrs. After overnight staining in Alizarin red solution (50 mg/l Alizarin red in 2% KOH), skeletons were cleared in the following ratios of 2% KOH to glycerol; 80∶20, 60∶40, 40∶60 and indefinitely stored in 20% KOH/80% glycerol. Photographs were taken by using a dissecting microscope [64].

### iPond

1.5×10^8^ iMEFs were incubated with 10 µM EdU (Invitrogen) for various time periods. For thymidine chase experiments, EdU labeled cells were washed once with pre-equilibrated (temperature, pH and thymidine) medium and then incubated with 10 µM thymidine for various times. After labeling and/or pulse-chase, cells were cross-linked with 1% formaldehyde/PBS for 20 min, quenched with 1.25 M glycine and scraped off the plates and collected. After washing three times with PBS, the cell pellet was then resuspended in 0.25% Triton-X 100/PBS to permeabilize in room temperature. Cells were spun down after permeabilization and washed first with 0.5% BSA/PBS and then with PBS. Cells were incubated with either click reaction buffer (10 µM biotin azide, 10 mM sodium ascorbate, 2 µM CuSO_4_ in PBS) or control buffer (as reaction buffer but DMSO added instead of biotin azide) at a concentration of about 3×10^7^ cells/ml for 1 hr at room temperature, protected from light. After incubation, cells were again washed with 0.5% BSA/PBS and PBS. Cells were then lysed with lysis buffer (1% SDS, 50 mM Tris pH 8.0, 1 µg/ml Leupeptin, 1 µg/ml aportinin) at a concentration of 1.5×10^7^ cells/µl. Samples were then sonicated using the Bioruptor (Diagenode) for 30 sec on and 60 sec off per cycle for 12 cycles. Samples were then spun down and supernatant was filtered through 90 micron nylon mesh (Small Parts) and diluted with PBS containing protease inhibitors. An aliquot of the lysate was kept as input, the rest was incubated with prewashed Streptavidin-agarose beads (Novagen) for 16 hrs at 4°C. The beads were then washed once with lysis buffer, once with 1 M NaCl, and twice with lysis buffer. Beads were boiled with 2× SDS dye for 25 min at 95°C. Proteins were resolved by SDS-PAGE and detected by western blot. Antibodies used in this study include: PCNA (Santa Cruz Biotechnology), H3 K9Ac (Abcam), H3 K14Ac (Abcam), H3 K18Ac (Upstate), H3 K23Ac (Upstate), H3 K27Ac (Upstate), H4 K5Ac (Abcam), H4 K12Ac (Abcam).

### Analysis of newly synthesized histones

For the electrophoretic analysis of newly synthesized histones, cultured MEF cells were pulse-labeled with 80 µCi/ml [^3^H]lysine (PerkinElmer Life Sciences) for 12 min, as described previously [65]. To inhibit histone deacetylation, labeling was performed in the presence of 50 mM sodium butyrate and 1 µM Trichostatin A. Acid-soluble nuclear proteins were prepared according to published protocols [66]. Fluorography of labeled histones after separation in acid-urea polyacrylamide gels was performed as described previously [67], [68].

## Supporting Information

Figure S1 Hat1^−/−^ embryos display early defects in lung development. The histological appearance of lungs isolated from 11.5 d.p.c. Hat1^+/−^ and Hat1^−/−^embryos. Hat1^+/−^ embryos were used because the Hat1^+/+^ embryos morphology was disrupted during analysis. The Hat1^+/−^ embryos do not display any viability defects at birth. Staining was with hematoxylin eosin (H+E), α­Hat1 and α­Ki67 antibodies (as indicated). Magnification was 20×. Hat1 and Ki67 staining was quantitated with Histoquest software.
(EPS)

Figure S2 Growth rates of immortalized MEFs. Immortalized Hat1+/+ and Hat1^−/−^ MEFs were seeded in wells (2000 cells/well). At the indicated time points, viable cells were measured by MTT assay.
(EPS)

Figure S3 Hat1^−/−^ cells display increased levels of γH2AX. Immortalized Hat1^+/+^ and Hat1^−/−^ MEFs were treated with 50 mJ UV and then aliquots were harvested at the indicated times. Whole cell extracts were prepared and analyzed by Western blot probed with the indicated antibodies.
(EPS)

Figure S4 Genomic instability in Hat1^−/−^ MEFs. Metaphase spreads were generated from Hat1^−/−^ MEFs. A) Metaphase spreads showing examples of chromatid breaks and chromosome fusions (marked by arrows). B) Metaphase spreads showing examples of aneuploidy and tetraploidy.
(EPS)
